# Histological Analysis of Bone Repair in Mandibular Body Osteotomy Using Internal Fixation System in Three Different Gaps without Bone Graft in an Animal Model

**DOI:** 10.1155/2019/8043510

**Published:** 2019-07-24

**Authors:** Sergio Olate, Bélgica Vásquez, Cristian Sandoval, Adriana Vasconcellos, Juan Pablo Alister, Mariano del Sol

**Affiliations:** ^1^Division of Oral, Facial and Maxillofacial Surgery, Universidad de La Frontera, Temuco, Chile; ^2^Center for Excellence in Morphological and Surgical Studies (CEMyQ), Universidad de La Frontera, Temuco, Chile; ^3^Faculty of Health Sciences, Universidad de Tarapacá, Arica, Chile; ^4^PhD Program in Morphological Sciences, Faculty of Medicine, Universidad de La Frontera, Temuco, Chile; ^5^Faculty of Medicine, Universidad de La Frontera, Chile

## Abstract

The aim was to analyze histologically the bone repair in a mandibular osteotomy model with different gaps between the segments. Nine male rabbits who underwent osteotomies on the mandibular body were fixed with a 1.5 system plate and no bone graft; group 1 (2 mm gap between segments), group 2 (5 mm gap between segments), and group 3 (8 mm gap between segments) were included. After 8 weeks they were euthanized and the sample was processed for histological analysis. Group 1 showed advanced bone repair with cartilaginous tissue and cancellous bone, showing osteoblasts and type III collagenous fibers. In group 2, a more delayed ossification was observed, with an extensive area of peripheral ossifying cartilage and chondrocytes in greater number at the center of the defect; group 3 showed no evidence of ossification with fibrous tissue, a very low level of chondrocytes, and some bone sequestrate. We can conclude that, in this animal model, 2 or 5 mm gap in the osteotomy could be repaired as bone when fixation is used. The size of the gap is an important factor for the use of bone grafts considering endochondral ossification. This model can be used for graft analysis and related technologies.

## 1. Introduction

Bone repair in fractures and osteotomies includes a cascade of events that must interact adequately and sequentially to obtain a functional recovery of the system, the aim of which is to restore the continuity of the interrupted bone. Therefore, to understand and manage the factors that intervene in the bone consolidation of fractures and those that limit it is of great relevance to determining the best treatment options.

When a fracture is managed in conditions of optimal stability by means of anatomical reduction and interfragmentary compression, the bone repair occurs by primary or direct ossification, without the formation of a periosteal callus [1]. However, when the fracture or osteotomy is not completely stabilized and there is a gap or mobility between fragments, a secondary or indirect ossification occurs, which is the most common form of repair [2]. If this mobility exceeds the tolerance to the deformation of the tissue present in the callus, alterations to the consolidation will occur, such as pseudoarthrosis or hypertrophic nonunion [3]. Secondary repair involves the classic stages of injury, inflammation, repair (with the formation of a soft cartilaginous primary callus and then its mineralization), and bone remodeling, and this repair process involves a cartilage group that is replaced by bone [4]. The speed of consolidation is influenced by the fractured bone, the type of fracture, the treatment method, and the general state of the patient and his age [5, 6]. Local factors are also influential, like the separation of the bone ends, which could be related to bone loss or resorption at the fracture site, soft tissue interposition between the bone ends, and excessive traction or the use of internal fixation [7, 8].

These observations have determined that bone grafts and reconstructive techniques are needed when a bone defect appears with larger gaps, turning it into a critical defect [9, 10]. Different materials have been used to improve the condition of bone repair, whether they are bone grafts or a scaffold, with controversial results and without even obtaining a model that makes it possible to study the suitable repair of these tissues [11, 12]. Recognizing the morphological parameters involved in the bone repair of these defects can contribute to the knowledge and the orientation to determine the best reconstructive options in these defects. Thus, the aim of this study was to analyze histologically the bone repair at the site of the osteotomy of the rabbit mandibular body using an internal fixation system with three different gaps between the bone ends, without using bone grafts.

## 2. Materials and Methods

A descriptive study was conducted to identify the bone repair in mandibular defects in rabbits treated with osteosynthesis from a histological point of view.

### 2.1. Animals

Nine adult male rabbits (*Oryctolagus cuniculus*) were used, clinically healthy, and obtained from the vivarium of the Center of Excellence in Morphological and Surgical Studies (CEMyQ) at the Universidad de La Frontera, Chile. The experiments were conducted according to the instructions in the Guide for the Care and Use of Laboratory Animals [13]. The rabbits were kept in controlled environmental conditions in terms of temperature, environmental noise, and a cycle of 12 hours light/12 hours darkness. They had free access to food (pellets) and water throughout the experiment. The protocol for housing, care, and experimentation was approved by the Scientific Ethics Committee of the Universidad de La Frontera, Temuco, Chile. The rabbits were inspected daily by the animal healthcare personnel, who were trained to record each and every incident that might have occurred.

### 2.2. Surgical Procedure

To perform the surgery, the animals were anaesthetized with intramuscular ketamine (22 mg/kg) and xylazine (3 mg/kg) [14]. The surgery began with a trichotomy of the area. Then, a 2 cm long incision was made with a surgical blade (n° 15) in the right submandibular region along with a submandibular divulsion as far as the mandibular body. Then the regional detachment was done to expose the animal's mandibular body. Next, the linear osteotomy was performed on the mandibular body using a straight bur 2 mm in diameter, mounted on a low-speed motor (20,000 rpm). Before making the osteotomy, the selected plate was installed in the mandibular body as a template and after that the osteotomy was done laterally to medially to achieve total separation of the mandible; the selected area for the osteotomy was posterior to the last mandibular molar.

**Figure 1:**
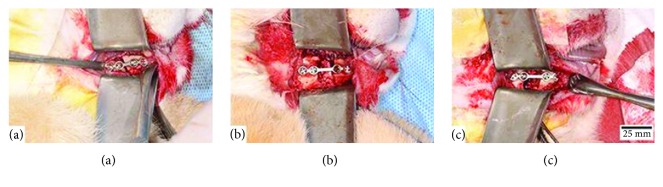
Linear osteotomy with bur in the mandibular body of a rabbit (*Oryctolagus cuniculus*). Internal rigid fixation osteosynthesis plate with 1.5 system (Jeil Medical Co., Seoul, Korea). (a) Group 1: 2 mm separation between the segments. (b) Group 2: 5 mm separation between the segments. (c) Group 3: 8 mm separation between the segments.

Then, the mandible was fixed and stabilized with an internal rigid fixation (1.5 system, Jeil Medical Co., Seoul, Korea), using a plate and four 5 mm monocortical screws in the same position as the template was installed. For this study, the separation used between the segments defined three groups: group 1 had a 2 mm separation between fragments, group 2, a 5 mm separation, and group 3, an 8 mm separation (Figure 1).

The animals were monitored and administered antibiotics and analgesia according to defined protocols [15]. After 8 weeks, they were euthanized and the samples were extracted for histological study. Euthanasia was performed with an overdose of intramuscular ketamine and xylazine.

### 2.3. Histological Analysis

The mandibular fragment that displayed the area of the osteotomy line was extracted, maintaining 4 mm as a safe distance from the anterior and posterior level of the osteosynthesis plate. The samples were fixed in 10% buffered formalin, pH 7.4, for 48 hours, decalcified in 10% EDTA buffer solution for a period of two months, dehydrated in a battery of ascending alcohols and embedded in paraffin (Histosec, Merck). Sections were made 3 *μ*m thick in a microtome (Microm HM 325, Thermo Fisher Scientific Inc., Waltham, MA, USA) and then stained with hematoxylin-eosin (HE) for the general analysis, Van Gieson's stain for the study of collagen fibers, HE plus Alcian blue stain (pH 2.5) to highlight cartilage, and Picrosirius red to analyze types I and III collagen fibers. The slides were analyzed in a Leica DM 750 microscope (Leica Microsystems, Germany) and photographed with a Leica ICC50 HD camera (Leica Microsystems, Germany).

## 3. Results

After surgery, the rabbits responded favorably to the intervention, returning to normal function with a standard diet within a maximum of 4 days. In the operation area, no immediate postoperative infection or surgical complications were observed or at the end of the study. The experimental design did not compromise the welfare of the animals, which allowed completing the course of the study successfully (8 weeks), without loss of animals. At the final stage, no significative loss weight was observed in the animals.

### 3.1. Group 1

**Figure 2:**
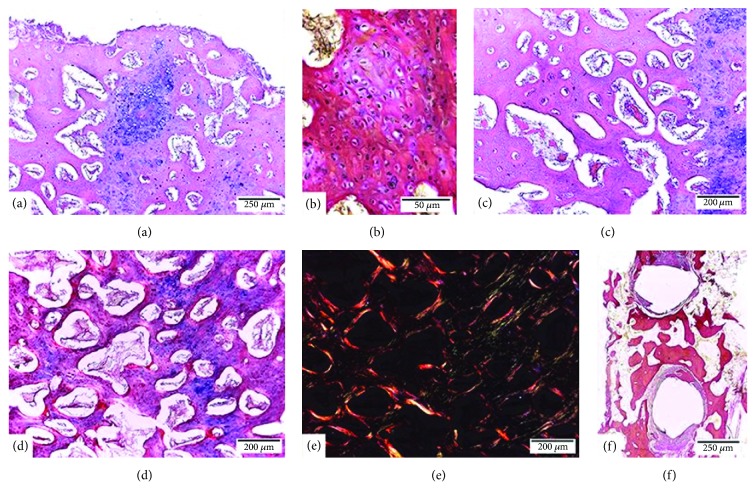
Bone repair of rabbit mandible (Oryctolagus cuniculus) after an osteotomy with 2 mm separation between the segments and an internal rigid fixation system 1.5. (a) Indirect consolidation of the mandible and formation of callus with endochondral ossification (HE). (b) Immature cancellous bone, fibrillar, shows abundant randomly distributed collagenous fibers (Van Gieson). (c) Mature cancellous bone, layered, shows more compact trabeculae whose fibers were organized concentrically to blood vessels (HE). (d) and (e) Areas with immature cancellous bone with greater cellularity and presence of type III collagen (green) and areas with mature cancellous bone with less cellularity and an increased presence of type I collagen (red and yellow) (Picrosirius red). (f) Peripheral fibrous reaction, without inflammatory elements in areas of contact with the screws (Van Gieson).

The area of the osteotomy line could be identified easily, without observing any bone interruption or separation. The histological analysis showed the formation of a cartilaginous callus, highlighting a process of almost complete indirect consolidation (Figure 2(a)).

The cartilaginous tissue was partially replaced by cancellous bone, and randomly placed collagen fibers were observed in its density (Figure 2(b)). However, in some areas fibers were noted that were organized in layers concentric to the blood vessels (Figure 2(c)). The medullary spaces were reduced and presented blood vessels and fibroblasts in their interior.

A large number of cells was observed in the immature areas, where osteoblasts and type III collagen fibers predominated, whereas, in the mature areas, less cellularity was observed, and osteocytes and type I collagen fibers predominated (Figures 2(d) and 2(e)). In the area with the screws, a peripheral fibrous reaction was observed without inflammatory elements (Figure 2(f)).

### 3.2. Group 2

**Figure 3:**
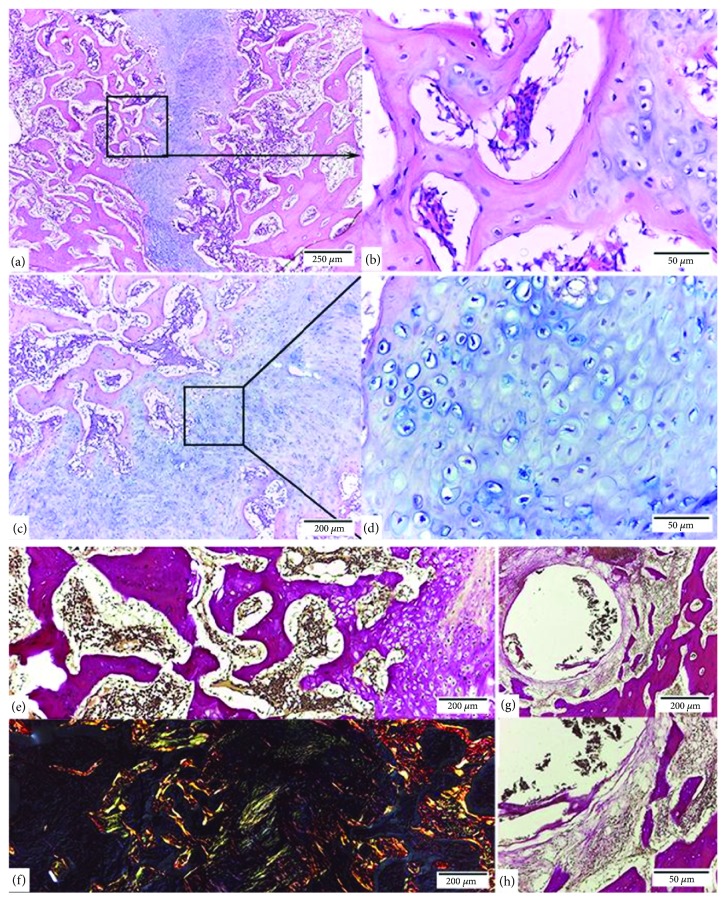
Bone repair of rabbit mandible (*Oryctolagus cuniculus*) after an osteotomy with 5 mm separation between the segments and internal rigid fixation system 1.5. (a) Cartilaginous callus on the osteotomy line (HE). (b) Area peripheral to the callus shows trabeculae in formation whose cartilaginous components have been replaced by bone tissue by means of endochondral ossification (HE). (c) and (d) In the cartilaginous callus, active chondrocytes immersed in a matrix positive to Alcian blue staining (HE, Alcian blue, pH 2.5) are observed. (e) Cancellous bone tissue next to the callus shows wide medullary spaces with abundant fibroblasts and blood vessels (Van Gieson). (f) Type III collagen (green) mainly in the cartilaginous tissue matrix and in the trabeculae peripheral to the callus (Picrosirius red). (g) and (h) Peripheral fibrous reaction, without inflammatory elements in areas of contact with the screws (Van Gieson).

There was a process of indirect consolidation at a more delayed stage of ossification than in group 1. The area of the osteotomy line was easily detected, showing an extensive cartilaginous area, the ossification of which was only peripheral, there being some outlines of newly formed trabeculae (Figures 3(a) and 3(b)). The cartilage matrix displayed little calcification in the central area and peripherally foci with active chondrocytes were observed (Figures 3(c) and 3(d)).

The bone tissue peripheral to the callus had characteristics of mature bone with thin layered trabeculae surrounded by lining cells. The medullary spaces next to the fracture were wide and invading the cartilaginous tissue (Figure 3(e)).

Type III collagen was present mainly in the cartilaginous tissue matrix and in the peripheral areas of the callus. However, type I collagen was observed essentially in the trabeculae of the mature bone tissue (Figure 3(f)). In the area with the screws, a peripheral fibrous reaction without inflammatory elements was also observed. The bone tissue next to this area exhibited histological characteristics of mature bone (Figures 3(g) and 3(h)).

### 3.3. Group 3

**Figure 4:**
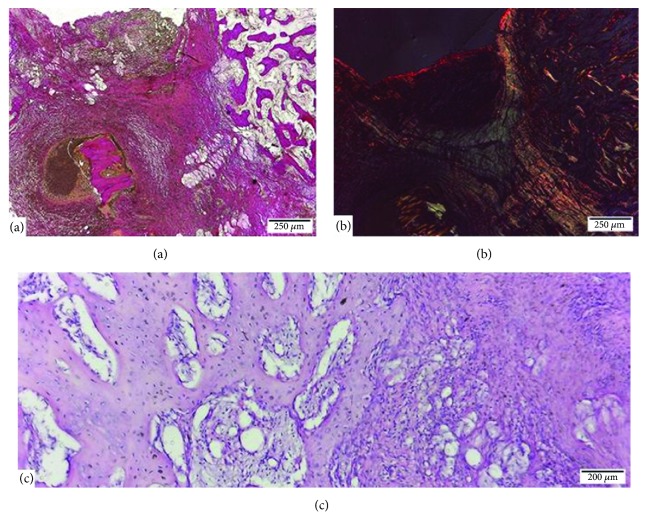
Bone repair of rabbit mandible (*Oryctolagus cuniculus*) after an osteotomy with an 8 mm separation between the segments and fixation with an internal rigid system 1.5. (a) The surface area of the osteotomy showed inflammation and presence of granulation tissue with abundant blood vessels, macrophages, fibroblasts, and lymphocyte cells (Van Gieson). (b) The deep area of the osteotomy was characterized by the presence of fibrous tissue (Picrosirius red). (c) The bone peripheral to the repair site displayed trabeculae of greater cellularity and thickness (HE).

No ossification was noted. The histological analysis showed that the repair of the fracture was being done based on the fibrous tissue. Under the periosteum, an inflammatory area with granulation tissue with abundant blood vessels, macrophages, fibroblasts, and lymphocyte cells predominated (Figure 4(a)). More deeply, fibroblastic proliferation with abundant collagen fiber production was observed (Figure 4(b)). In some samples the presence of partially digested bone sequestra could be seen. The bone peripheral to the repair site showed signs of reactivity to the inflammatory process (trabeculae with greater cellularity and thickness) (Figure 4(c)). As in the other groups, the tissue surrounding the screw showed peripheral fibrous reaction without inflammatory elements.

## 4. Discussion

In the facial skeleton, bone repair of fractures and osteotomies is mostly produced by (indirect) endochondral processes on the basis of the bone callus formation, generated from the periosteum and endosteum, which includes an initial inflammatory phase, cartilage formation, and subsequent bone mineralization and remodeling [4]. This type of ossification first demands the presence of chondrocytes; the hypertrophic chondrocytes present in the area express high levels of phosphatase alkaline and noncollagen proteins like osteonectin, osteopontin, osteocalcin, and bone sialoprotein involved in the mineralization of the cartilage matrix [16, 17]. Direct bone repair is less frequent, since it requires a perfect reduction and the compression of the fracture with a distance between the bone segments of less than 0.1 mm [18]. In addition, movement at the fracture site must be minimal, since direct consolidation of the bone requires rigid immobilization to enable the fragile medullary vessels to penetrate through the necrotic bone and penetrate the fracture. In this type of ossification, the necrotic bone is reabsorbed by osteoclasts to later be invaded by osteoblasts that synthesize lamellar bone directly [19].

The results of this research highlight the importance of the position and condition of the bone segments when the repair process from a fracture or osteotomy begins. The distance of the bone ends defined the type of repair and the evolutionary stage of the repair process. In groups 1 and 2 the repair was endochondral (indirect) and in group 3 (distance between the bone ends of 8 mm) there was no fracture consolidation; the gap was comprised mainly of fibrous tissue with abundant fibroblasts, without osteoplastic integration. On the same hand, in groups 1 and 2 bone formation in the lateral area of the defect was observed clinically, maybe related to the contact of this area with the periosteal tissue, as related by Alister et al. [20]; in the 8 mm bone gap this bone repair was not observed.

There are several factors that can cause a delay in consolidation; some are inherent to the patient, others to the fracture itself, and others to the medical management of the patient. However, inadequate and interrupted immobilization and the excessive separation of bone fragments are factors that can not only slow down the process but also cause pseudoarthrosis [21]. The histological analysis of our study showed that the timing of the consolidation was different in each group. Group 1 was in a more advanced stage of the consolidation process than groups 2 and 3, and the cartilaginous callus revealed an almost complete mineralization process. Group 2 also showed a cartilaginous callus in the gap between fragments; however, the mineralization of the callus was less evident than group 1. In group 3, the excessive separation of bone fragments caused the formation of a fibrous scar, without achieving any consolidation at 8 weeks after surgery.

Different models for studying bone repair processes have been reported. Sverzut et al. [22] conducted an investigation on dogs to study the repair response in linear osteotomies of the mandibular body stabilized with polymer-based osteosynthesis plates and screws. They identified clear differences between the newly formed and mature bone tissue. They reported that two weeks were not enough to get bone, but later, at 8 weeks, they did observe defined signs of repair with areas of cortical bone remodeling due to reactions by the periosteum and endosteum. Our study defined an economical and easily maintained animal model, identifying that it is possible to obtain adequate repair in 2 mm gaps between bone fragments at 8 weeks, without alterations in their repair process. In this sense, it is important to note that small rodents do not have osteons, whereas rabbits, goats, and dogs are in fact present, which is an important advantage in terms of extrapolating the results to the human condition [23].

Historically, studies on bone repair associated with hypertrophic chondrocytes have focused on the secretion of alkaline phosphatase and the presence of angiogenic signals that caused calcification of the cartilage matrix and vascular invasion [24]; the apoptosis of hypertrophic chondrocytes corresponded to the areas of cartilage in the degeneration phase, being associated temporally and spatially with the mineralization of cartilage removed by osteoclasts [25]. The cavities produced by the death of the chondrocytes were later filled by osteoprogenitor cells that used this base matrix as a scaffold that secretes osteoids, forming the nonmineralized phase of the cancellous and cortical bone [24]. The matrix that surrounds the hypertrophic cartilage was also mineralized by the hydroxyapatite deposition, depending on the presence of alkaline phosphatase [26].

On the other hand, some studies show that the hypertrophic chondrocyte can modify and even transform into cells like osteoblasts and osteocytes [27, 28], so that some chondrocytes (light or dark, for example) can present differences in their gene expression patterns [29]. Therefore, many cell signaling that includes bone morphogenetic proteins (BMPs) (with the opposite effect to fibroblast growth factors (FGFs) in the chondrocyte differentiation), FGFs, Wnt (associated with cell differentiation in osteoblasts), insulin-like growth factors (IGFs), and some Hedgehog proteins are recognized for their importance in bone repair [30].

It is interesting to note in our results that the presence of chondrocytes in the group with the largest gap between bone fragments (group 3) was very small in relation to the inflammatory and fibroblast cells that were present in the repair tissue. In group 1, the chondrocytes were active and present across the regeneration line, establishing a positive bone repair. Bear in mind that cartilage is not a vascularized tissue, so a large part of the signals for the chondrocytes to function must be synthesized locally or sent from adjacent tissues to the matrix [31]; therefore, the matrix plays an important role in regulating and delivering products that determine the maturation or apoptosis of chondrocytes and acting as a scaffold for osteoblast progenitors [32]. The formation of mineralized tissue around the cartilage can be a critical event for the onset of apoptosis of the cartilaginous cells. In this sense, hypertrophic chondrocytes adjacent to the perichondrium can be transformed into osteoblasts and form a ring around the central area of the developing bone, expressing high levels of vascular endothelial growth factor (VEGF). This growth factor can also act as a chemotactic for the invasion of osteoclasts, endothelial vascular cells, and hematopoietic progenitors inside the hypertrophic cartilage that is related to the perichondrium and periosteum, promoting bone repair [33].

Gerber et al. [34] demonstrated that VEGF has a fundamental role in growth and differentiation in endochondral ossification. VEGF is produced by hypertrophic chondrocytes, recruiting endothelial cells to induce and maintain blood vessels; this determines that the presence of osteoblasts and progressive neovascularization of the area are necessary for endochondral bone formation [35]. The stability of segments by fixation and the size of the defect, therefore, must be sufficient to promote the formation of cartilaginous tissue, in which the matrix can contain and offer a suitable environment for chondrogenic function. VEGF is said to regulate the formation of blood vessels and early stages of osteoblast differentiation during perichondrial maturation; therefore, the presence of blood vessels may also be an indicator of the state of the bone repair [36]. In fact, VEGF produced by bone cells help in the homeostasis of the bone, promoting the differentiation to osteoblasts and not to bone adipocytes like those observed in the bone trabeculae [33].

Bone repair in defects of the facial skeleton is subject to the distance between the bone segments, stability of the segments (immobilization), and environmental conditions (infection, presence of tissues like periosteum, among others) [37]. Our study identified that, regardless of the size of the defect, in groups 1 and 2 the type of cells and shape of the matrix followed a similar repair pattern; however, there were differences in the stage of advancement of the bone repair.

Based on our observations, it may be concluded that, under adequate stabilization with osteosynthesis plates, the distance between bone segments is a relevant factor in bone repair; the animal model used in this study is useful for analyzing the response of bone grafts in mandibular reconstructions.

## Data Availability

The data used to support the findings of this study are available from the corresponding author upon request.
